# Pembrolizumab in gestational trophoblastic neoplasia: Systematic review and meta-analysis with sub-group analysis of potential prognostic factors

**DOI:** 10.1016/j.clinsp.2025.100583

**Published:** 2025-03-02

**Authors:** Marcio Barcellos, Antonio Braga, Matheus Machado Rech, Solange Artimos de Oliveira, Jose Mauro Madi, Sue Yazaki Sun, Jorge de Rezende-Filho, Kevin M. Elias, Neil S. Horowitz, Ross S. Berkowitz

**Affiliations:** a Centro de Doenças Trofoblásticas do Rio de Janeiro, Maternidade Escola da Universidade Federal do Rio de Janeiro, Rio de Janeiro, RJ, Brazil; b Postgraduate Program in Medical Sciences, Faculdade de Medicina, Universidade Federal Fluminense, Rio de Janeiro, RJ, Brazil; c Postgraduate Program in Applied Health Sciences, Universidade de Vassouras, Vassouras, RJ, Brazil; d Centro de Doenças Trofoblásticas de Caxias do Sul, Faculdade de Medicina, Universidade de Caxias do Sul, Caxias do Sul, RS, Brazil; e Department of Obstetrics, Escola Paulista de Medicina, Universidade Federal de São Paulo, São Paulo, SP, Brazil; f New England Trophoblastic Disease Center, Division of Gynecologic Oncology, Department of Obstetrics, Gynecology and Reproductive Biology, Brigham and Women's Hospital, Dana-Farber Cancer Institute, Harvard Medical School, Boston, MA, USA

**Keywords:** Gestational trophoblastic neoplasia, Choriocarcinoma, Immunotherapy, PD-1/PD-L1 inhibitors, Pembrolizumab

## Abstract

**Objective:**

To assess the performance of pembrolizumab for the treatment of Gestational Trophoblastic Neoplasia (GTN).

**Methods:**

The Medical Subject Headings related to immunotherapy/pembrolizumab and GTN were used alone or in combination to retrieve relevant articles. The authors searched in EMBASE, MEDLINE/PubMed, Elsevier's Scopus, and Web of Science until November/2024. The authors included any randomized controlled trials, cohort studies, case series, and case reports focusing on pembrolizumab treatment in GTN. Meta-analysis of proportions was carried out employing a random-effects model. The meta-analysis employed the inverse variance method, with the arcsine link function for the analysis of proportional data. All analyses were performed using Stata 18. For all analyses, a p-value < 0.05 indicated statistical significance. This study was registered on PROSPERO (CRD42023493329).

**Results:**

A total of 550 studies were identified after a literature search among which 15 original studies were included in the systematic review and in the meta-analysis. Pembrolizumab induced complete sustained remission in 71.59% (95% CI 53.27‒84.78%; I^2^ = 0.00%, H^2^ = 1.00, p = 0.90) of cases. The subgroups meta-analysis showed pembrolizumab had similar performance, regardless of age (< 40 vs. ≥ 40-years-old, p = 0.38), GTN histopathology (Placental Site Trophoblastic Tumor [PSTT], Epithelioid Trophoblastic Tumor [ETT]/noninvasive mole/others versus invasive mole/choriocarcinoma, p = 0.48), time from diagnosis to the beginning of immunotherapy (< 4 vs. ≥ 4-years, p = 0.84), pembrolizumab combined with chemotherapy (yes vs. no, p = 0.66).

**Conclusions:**

Pembrolizumab seems an effective treatment for patients with high-risk GTN with chemoresistant or relapsed disease, including cases of PSTT/ETT, notwithstanding patient age, time to initiate immunotherapy and whether or not it was associated with chemotherapy.

## Highlights

• Pembrolizumab is effective for GTN multi-drug resistant treatment.
• Pembrolizumab seems also effective for PSTT/ETT treatment.
• Pembrolizumab efficacy is similar in cases of resistance or GTN relapse.

## Introduction

Gestational trophoblastic neoplasia refers to malignancies derived from the proliferative trophoblastic allograft, affecting approximately 20,000 women per year worldwide.^1^^,^^2^ The broad expression of paternal antigens on the cell surface of these tumors,^3^ combined with the presence of a biological marker that allows monitoring of treatment (human Chorionic Gonadotropin – hCG),^4^ allows for a high cure rate of GTN.^5^ Furthermore, the categorization of low- and high-risk GTN, according to the criteria of the International Federation of Gynecology and Obstetrics, allows treating most patients with lower toxicity regimens in cases at low-risk for chemoresistance, reserving chemotherapy containing multiple agents for high-risk GTN, in which there is an increased risk for resistance to monochemotherapy.^6^^,^^7^

Nevertheless, more advanced cases of GTN with multi metastatic presentation, chemoresistance, more aggressive histopathological forms (such as choriocarcinoma, Placental Site Trophoblastic Tumor – PSTT, Epithelioid Trophoblastic Tumor – ETT), delays in initiating chemotherapy, remote residence from a trophoblastic disease Reference Center (RC), or treatment initiation outside of an RC are associated with lethality from this disease.^8^^,^^9^ In these challenging situations, the development of new therapies is urgent and necessary to mitigate the risk of death for these patients.

The successful introduction of immunotherapy for solid tumors in gynecologic oncology has now extended to the treatment for GTN, especially in cases of high-risk and chemoresistance/relapse disease.^10^, ^11^, ^12^, ^13^ Among the immunotherapeutics reported for use in GTN, pembrolizumab has been the best described. Pembrolizumab acts by inhibiting programmed cell death Protein-1 (PD-1) receptors on lymphocytes, blocking the ligands that would otherwise deactivate effector T-cells and prevent an immunological response. Ubiquitous PD-L1 expression in the trophoblast,^14^ coupled with the allograft nature of the placenta, makes GTN an ideal therapeutic target for pembrolizumab.^12^

Although reports and small case series have presented encouraging results from the early international experience with pembrolizumab in GTN, it is essential not only to summarize the effectiveness of this treatment but also to delineate both areas of success and areas of uncertainty, especially identifying possible GTN profiles that may be unresponsive to this immunotherapy.

The aim of this systematic review and meta-analysis is to assess the overall effectiveness of pembrolizumab, as well as its performance in different scenarios where there is doubt as to whether this treatment is equally effective in GTN. There are doubts about whether the response to pembrolizumab is influenced by the patient's age, the histopathology of GTN, the number of previous lines of chemotherapy, or even the time of initiation of this treatment. Given the small case series and cohorts reported to date, a meta-analysis of pembrolizumab is especially important because this treatment is considered one of the last opportunities to cure patients with GTN who no longer respond to any line of chemotherapy, yet there are still crucial doubts about the limits and possibilities for this new approach.

## Methods

### Design

The methodological approach to evidence searching and synthesis followed the Preferred Reporting Items for Systematic Review and Meta-Analysis Protocols (PRISMA) recommendations^15^ as well as the MOOSE (Meta-analyses of Observational Studies in Epidemiology) Checklist.^16^ This study was registered at PROSPERO (December 27, 2023), the International Prospective Register of Systematic Reviews, at the University of York (CRD42023493329).^17^

### Eligibility criteria for included studies

This review aimed to include Randomized Controlled Trials (RCTs), cohort studies, case series, and case reports focusing on pembrolizumab use in GTN. Studies were selected based on their relevance to the review questions, with no restrictions on study size or follow-up duration. Exclusion criteria included non-English language studies, animal studies, and abstracts or presentations without full-text availability (e.g., conference abstracts, and posters).

For systematic review and meta-analysis of studies on risk factors and prognosis, that respond to the question: *What is the efficacy of pembrolizumab in high-risk gestational trophoblastic neoplasia* the acronym PICO was used, which corresponds to the areas P (population), I (intervention), C (comparison) and O (outcome):

1. Population: Women who had high-risk GTN (both in cases of first-line treatment and in cases of chemoresistance or relapse, as well as PSTT or ETT);
2. Intervention: Immunotherapy with pembrolizumab;
3. Comparison: Resistance to pembrolizumab with disease progression and/or death;
4. Outcome: Remission after immunotherapy (attested by 3-weekly hCG levels < 5 IU/L, or in cases of PSTT/ETT, also the radiographic resolution of disease).

### Search methods for identifying studies

The keywords and Medical Subject Headings related to immunotherapy/pembrolizumab and GTN, used alone or in combination (and with synonyms and closely related words) to retrieve relevant articles: (("gestational trophoblastic disease" [All fields]) OR ("gestational trophoblastic neoplasia" [All fields])) ("choriocarcinoma" [All fields]) OR ("placental site trophoblastic tumor" [All fields]) OR ("epithelioid trophoblastic tumor" [All Fields])) AND ("immunotherapy" [MeSH Terms]) AND (("remission" [All fields]) OR ("persistent" [All fields]) OR ("progression")) AND (("chemoresistant") OR ("refractory" [All fields]) OR ("resistant"[All fields]) OR ("non-respondent" [All fields]) AND ((“relapse” [All fields]) OR (“recurrence” [ All fields])). The authors searched in Excerpta Medica Database (EMBASE), Medical Literature Analysis and Retrieval System Online (MEDLINE)/PubMed, Elsevier's Scopus and Web of Science up to November 28th, 2024. The authors also screened reference lists of relevant studies and reviews for additional articles. There was no publication year restriction. The search strategy developed for each database are available in the supplemental files (Supplemental Table 1).

### Data collection

Two independent researchers (AB and JMM) evaluated all titles and abstracts for the initial screening of the studies. A third author adjudicated any discrepancy (SYS). All selected articles were read in full to assess the eligibility of the studies according to the inclusion and exclusion criteria to be considered in the systematic review. The researchers extracted all data from the retrieved articles, independently, using a standardized data extraction sheet.

In case of duplicate publications and more than one publication of a preliminary study, the authors attempted to maximize the use of information by simultaneous evaluation of all available data but did not include the same group of patients more than once in the analysis.

The following information was extracted for each study (when available):

1. Study characteristics: title, author, year of publication, country, sample size;
2. Population characteristics: age, the origin of GTN, histopathology form, time to initiate immunotherapy, FIGO score, hCG level pre-immunotherapy, immunotherapy combined with chemotherapy, indication for immunotherapy, number of previous chemotherapy lines, and number of consolidations cycles.
3. Outcomes: remission or progression/death.

### Quality and evidence assessment

The quality assessment was performed using the Newcastle Ottawa scale for case series and case reports that can be categorized into four domains: selection, ascertainment, causality, and reporting.^18^ These four domains with leading explanatory questions are summarized in Supplemental Figure 1. Two independent researchers assessed the quality and the evidence (AB and JMM), independently, a third author adjudicated any discrepancy (SYS).

Likewise, the quality assessment was performed using the Grading of Recommendations Assessment, Development and Evaluation (GRADE) to rate the quality of the body of evidence retrieved.^19^ Two independent researchers assessed the quality and the evidence (AB and SYS); independently, a third author adjudicated any discrepancy (JMM).

### Statistical analysis

Meta-analysis of proportions was carried out employing a random-effects model, as previously described.^18^^,^^20^ Heterogeneity was quantified using restricted maximum likelihood as the variance estimator. The meta-analysis employed the inverse variance method, with the arcsine link function for the analysis of proportion data. A continuity correction of 0.5 was adopted in studies with zero cell frequencies (only used to calculate individual study results) to avoid computational issues. All analyses were performed using Stata 18. For all analyses, a p-value < 0.05 indicated statistical significance.

Heterogeneity among the included studies was assessed using the I^2^ and H^2^ statistics. Even though there is no universal rule to grade heterogeneity based on H^2^, I^2^ values show a good correlation with the heterogeneity of studies and should be interpreted as follows: up to 40%, might not be important; 30% to 60%, moderate heterogeneity; 50% to 90%, substantial heterogeneity; and 75% to 100%, considerable heterogeneity.^21^

The authors anticipated that most of the cases would be case reports and case series. For this analysis, the authors followed Murad et al. recommendations for case reports/case series meta-analysis.^18^ Although less common, this method, with minor variations, has been reported in extensive literature, while the performance of meta-analysis of proportions could be an alternative approach to deal with the absence of clinical trials.^20^

### Subgroup analyses

Factors collected for the included studies were used to stratify groups in subgroup analyses, supporting the investigation of potential sources of heterogeneity or inconsistencies. These factors included age, origin of GTN, histopathology forms of GTN, number of previous chemotherapy lines, indication for immunotherapy, time to initiate immunotherapy, immunotherapy combined with chemotherapy, and number of consolidation cycles. The rationale for selecting these factors was based on their potential clinical relevance and impact on treatment outcomes.

### Sensitivity analysis

The authors conducted a sensitivity analysis using a leave-one-out approach to check the robustness of the results while iteratively removing one study at a time. This approach allowed us to assess the influence of individual studies on the overall pooled estimate and determine if the findings were heavily dependent on any single study. Potential limitations of this approach, such as reduced statistical power, were considered when interpreting the results.

## Results

### Systematic review

**Fig. 1:**
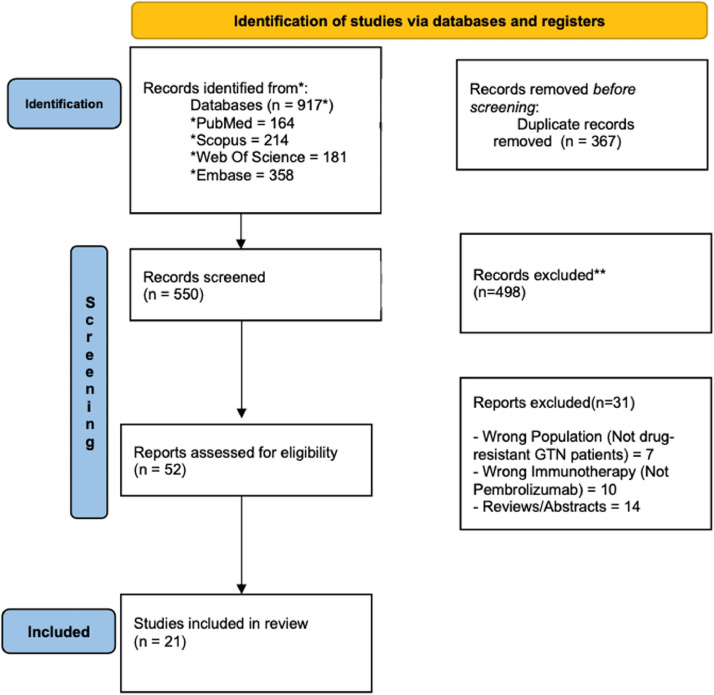
Prisma 2009 Flow Diagram.

The study selection process is illustrated in the PRISMA flow diagram (Fig. 1). A total of 15 studies, presented in Table 1, including 21 patients' individual data and 18 remission reports, were included in the meta-analysis.^10^^,^^13^^,^^22^, ^23^, ^24^, ^25^, ^26^, ^27^, ^28^, ^29^, ^30^, ^31^, ^32^, ^33^, ^34^

**Table 1:** Characteristics of the studies and clinical and oncologic outcomes of patients treated with pembrolizumab for Gestational Trophoblastic Neoplasia (GTN) included in this study.

Study	Year	Country	Remission	Age	Origin of GTN	Histopathology	Time to initiate Im (weeks)	FIGO score	hCG pre Im	Im + Ch	Indication for Im	Number of previous Ch lines	Number of consolidation cycles
Ghorani et al.	2017	UK Sweden	Yes	42	‒	Choriocarcinoma	36	17	80	No	Relapse	6	5
			Death	52	‒	Mixed PSTT^6^/ETT^7^	192	8	2468	No	Relapse	2	*
			Yes	48	‒	PSTT^6^	132	20	73	No	Resistance	4	5
			Yes	37	‒	Choriocarcinoma	7	6	118	No	Relapse	6	5
Huang et al.	2017	US	Yes	26	AD	Choriocarcinoma	6	18	700	No	Resistance	3	2^a^
Choi et al.	2019	South Korea	Yes	39	‒	PSTT	37	15	67	No	Resistance	3	13
			Yes	26	‒	ETT	73	14	57	No	Resistance	2	4^b^
Clair et al.	2020	US	Yes	30	CHM	Choriocarcinoma	35	1	4527	No	Relapse	6	Not reported
Goldfarb et al.	2020	US	Progression	50	‒	Choriocarcinoma	39	‒	480	No	Relapse	6	3
Pisani et al.	2021	Malta	Yes	49	AD	ETT	204	‒	0	No	First line	0	Not reported
Bell et al.	2021	US	Yes	47	‒	ETT	156	‒	9	No	Resistance	1	c
Porter et al.	2021	US/UK	Yes	34	AD	PSTT	7	‒	55	Yes	Resistance	2	Not reported^d^
Polnaszek et al.	2021	US	Yes	23	Abortion	PSTT	2	‒	440	No	First line	0	0^e^
Paspalj et al.	2022	Austria	Yes	31	AD	Choriocarcinoma	10	10	2115	No	Resistance	3	3
Wong et al.	2022	US	Yes	44	‒	Choriocarcinoma	‒	13	26	No	Relapse	3	f
Braga et al.	2023	Brazil	Yes	26	CHM	‒	22	5	17,000	No	Relapse	3	3
			Yes	29	CHM	Invasive mole	21	2	‒	No	Relapse	4	5
			Yes	41	Abortion	‒	14	8	‒	No	Resistance	3	3
Lehmann et al.	2023	Austria	Yes	32	AD	Choriocarcinoma	140	5	2598	Yes	Relpase	3	12
Niimi et al.	2023	Japan	Progression	38	Abortion	Choriocarcinoma	25	6	48,000	No	Resistance	6	*
Helbig et al.	2023	Germany	Yes	46	AD	ETT	11	‒	7	No	Relapse	1	Not reported

Im, Immunotherapy; FIGO, International Federation of Gynecologists and Obstetricians; hCG, Human Chorionic Gonadotropin (UI/L); Ch, Chemotherapy; AD, After Delivery; PSTT, Placental Site Trophoblastic Tumor; ETT, Epithelioid Trophoblastic Tumor.

⁎ Not applicable as there was no remission.

a Need to reduce the dosage of the 2 consolidation cycles of pembrolizumab in 50% due to toxicity.

b The institution's tumor board decided to continue treatment with pembrolizumab, even after remission.

c The authors did not specify the number of pembrolizumab cycles until remission and consolidation cycles. However, after 29 cycles of pembrolizumab, with remission for 12-months, the patient continued immunotherapy.

d Although the number of consolidation chemotherapy cycles with pembrolizumab was not reported, the authors reported that they used, in addition to pembrolizumab, 5 consolidation cycles with the EP/EMA regimen, replacing cisplatin to carboplatin in the last cycle due to toxicity (thrombocytopenia, ototoxicity and tinnitus). Finally, the authors reported that she is still on consolidation treatment with pembrolizumab.

e This patient was scheduled for 4 pembrolizumab consolidation cycles, which were not administered once the patient became pregnant.

f The patient achieves remission after 2 cycles of pembrolizumab (followed by 5 consolidation cycles). However, she relapsed after 6 months and was again treated with pembrolizumab. The report was unclear but suggested that the patient achieved remission after 4 further cycles of pembrolizumab, followed by 21 consolidation cycles. The patient was still undergoing consolidation chemotherapy at the time of publication of the case report, with no evidence of disease and with normal hCG levels after 2-months of the end of immunotherapy.

Supplemental Table 2 provides an overview of the sample results from the included studies. The mean age of the women studied was 38-years-old, with older maternal age among those who did not respond to immunotherapy (50-years-old), compared to those who achieved remission (35.5-years-old, p = 0.05). Choriocarcinoma was the most prevalent tumor histopathology (9/21%‒42.9%), although there was no significant difference associated with histopathology with respect to remission after immunotherapy. The median time to initiate immunotherapy was 35 days and, although the hCG level pre-immunotherapy was higher among those who did not respond to immunotherapy (2468 UI/L) compared to those who went into remission (38.1 IU/L), this difference was not significant (p = 0.07). Although there were 2 cases of pembrolizumab indicated as first-line treatment for high-risk GTN (9.52%), the vast majority were for treatment of chemoresistant (42.86%) or relapsed disease (47.61%).

### Meta-analysis

**Fig. 2:**
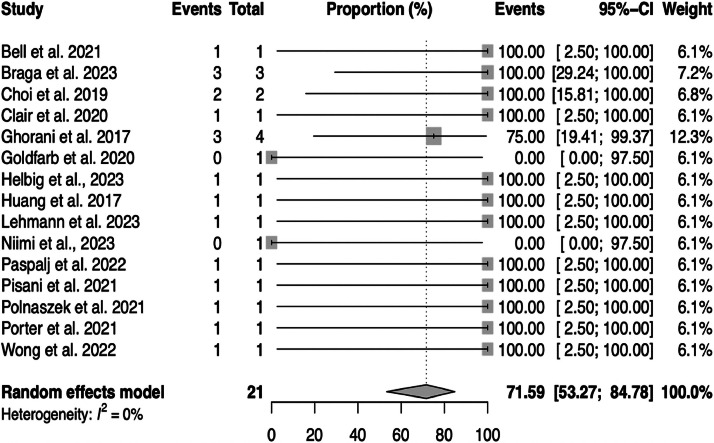
Forest plot and pooled proportion of patients with sustained remission after pembrolizumab for gestational trophoblastic neoplasia.

A meta-analysis was conducted to estimate the pooled proportion of GTN remission after pembrolizumab for the 15 studies included. The random-effects model yielded a pooled proportion estimate of 0.7159 (95% CI 0.53; 0.84), suggesting that the efficacy of pembrolizumab in the population of this study is approximately 71.59% (within the range of 53.27% to 84.78%), as can be seen in Fig. 2.

Heterogeneity among the included studies was assessed using several measures. The between-study variance (tau-squared) and its square root (tau) were both estimated to be 0, suggesting no observed between-study variance. The I^2^ statistic for inconsistency was 0.00%, and the H^2^ statistic was 1.00 (p = 0.90), indicating no observed heterogeneity among the studies included in the sensitivity analysis. This suggests that the variation in the estimated proportions was consistent with sampling variability rather than differences in study populations or interventions.

### Subgroup analysis

**Fig. 3:**
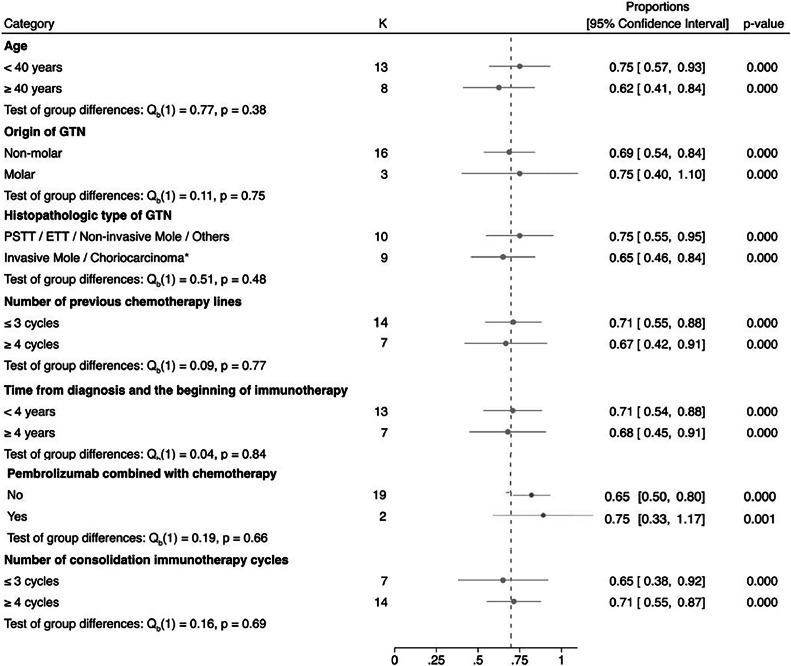
Forest plot and pooled proportion of various subgroups of factors that may influence the efficacy of pembrolizumab treatment.

The subgroups meta-analysis (Fig. 3) assessed the influence of various factors on the efficacy of pembrolizumab treatment, including age (< 40 vs. ≥ 40-years-old, p = 0.38), the origin of GTN (non-molar vs. molar, p = 0.75), histopathologic type of GTN (PSTT/ETT/noninvasive mole/others versus invasive mole/choriocarcinoma, p = 0.48), number of previous chemotherapy lines (≤ 3 vs. ≥ 4 lines, p = 0.77), time from diagnosis to the beginning of immunotherapy (< 4 vs. ≥ 4-years, p = 0.84), pembrolizumab combined with chemotherapy (yes vs. no, p = 0.66), and number of consolidation immunotherapy cycles (≤ 3 vs. ≥ 4 cycles, p = 0.69). This in-depth analysis found that these factors did not significantly affect treatment success, suggesting pembrolizumab's uniform effectiveness across different patient subgroups.

### Sensitivity analysis

**Fig. 4:**
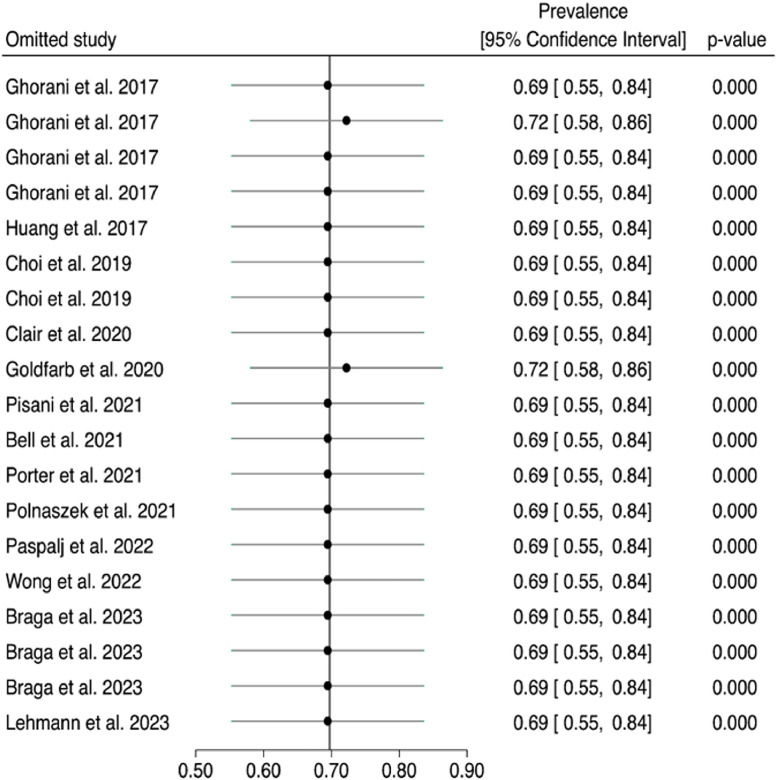
Forest plot and pooled proportion of patients with sustained remission after pembrolizumab considering the leave-one-out sensitivity analysis.

The leave-one-out sensitivity analysis (Fig. 4) indicated that the meta-analysis findings were robust and not overly dependent on any single study. The efficacy rate remained relatively stable, ranging from approximately 69% to 72% across different iterations, with all p-values indicating statistical significance. This finding suggests that the overall pooled estimate is not heavily influenced by any individual study and provides further confidence in the robustness of the results.

**Fig. 5:**
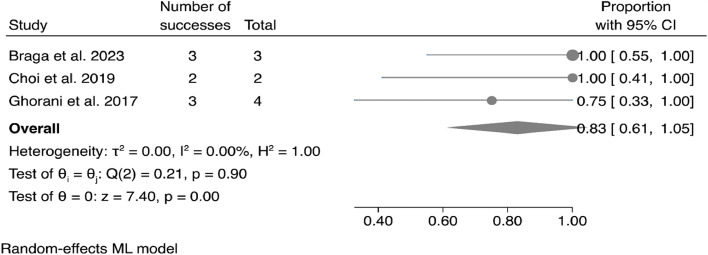
Forest plot and pooled proportion of patients with sustained remission after pembrolizumab considering only case series analysis.

The meta-analysis of case series data (Fig. 5) confirmed the primary analysis's findings, showing an overall success rate of 83% (95% CI 61%‒105%).

### Publication bias

Considering the quality assessment performed by the Newcastle Ottawa scale for case series and case reports,^18^ the authors observed that there was a low-risk of bias in 9/15 (60%) of the studies included in this meta-analysis, with none of the other studies having any score that signaled a high risk of bias in the analyzed domains (Supplemental Fig. 1).

Regarding the GRADE assessment,^19^ the overall quality of the evidence is considered low, as further research is very likely to have an important impact on confidence in the estimate of effect and may change the estimate. This classification is mainly due to the nature of the studies included in this meta-analysis composed mainly of case reports and small case series.

A funnel plot was generated to visually assess publication bias (Supplemental Fig. 2). Although some asymmetry was observed, further analysis using the linear regression test of funnel plot asymmetry provided additional insights. The test statistic (*t*) was -0.68, with 13 degrees of freedom (df) and a p-value of 0.50. The bias estimate was -0.94 with a Standard Error (SE) of 1.39, and the intercept was 2.40 with an SE of 2.17. These results indicate no statistically significant evidence of funnel plot asymmetry (p = 0.50). The multiplicative residual heterogeneity variance (tau^2^) was 0.28.

## Discussion

This meta-analysis indicates that pembrolizumab is effective in the treatment of high-risk GTN, with no differences in remission rates in cases of resistant or relapsed disease. The pooled proportion estimate of 71.59% for the efficacy of pembrolizumab in high-risk GTN is a key finding, as it provides evidence of the potential clinical utility of this immunotherapy in a challenging patient population. The low heterogeneity observed among the included studies further supports the consistency of this finding.

Likewise, the results of this study point to the effectiveness of pembrolizumab in cases of high-risk GTN, regardless of the patient's age, the histopathological type of GTN, or the previous number of lines of chemotherapy used. Furthermore, the good response to pembrolizumab was maintained notwithstanding the time to initiate immunotherapy and irrespective of whether it was simultaneously combined with chemotherapy or not.

Since the first reports of immunotherapy for the treatment of high-risk GTN were presented in the literature,^10^^,^^22^^,^^23^ most with successful outcomes, this innovative treatment has been the hope of a cure for women affected by this tumor, with less toxicity.^12^^,^^13^^,^^35^ Among the immunotherapeutics prescribed in GTN, pembrolizumab has been the most used,^10^^,^^13^^,^^22^, ^23^, ^24^, ^25^, ^26^, ^27^, ^28^, ^29^, ^30^, ^31^, ^32^, ^33^, ^34^ justifying its choice for this meta-analysis.

Although a previous systematic review reported a lower response to pembrolizumab in patients with advanced maternal age (over 40 years of age),^13^ the present meta-analysis, with the inclusion of new studies, did not report this difference. This data is especially important since advanced maternal age is often associated with GTN chemoresistance,^6^^,^^7^ even though more recent studies have not supported this association.^36^ In spite of the fact there are speculations about immunosenescence or even age-dependent responses to immunotherapy,^37^^,^^38^ the results of this meta-analysis are potentially encouraging for its use in GTN, regardless of the patient's age.

GTN is a highly chemosensitive disease with 80%‒90% of high‐risk cases attaining a cure.^8^^,^^35^^,^^39^^,^^40^ However, cases of high-risk GTN with chemoresistance or even relapse constitute a major therapeutic challenge. Notwithstanding the fact that cases of chemoresistance appear to have a more unfavorable prognosis than cases of relapse,^40^ both are treated with toxic chemotherapy regimens containing multiple agents.^1^^,^^35^^,^^39^ The favorable results of pembrolizumab both for cases of chemoresistance and relapse, establish a new opportunity for a cure for the most aggressive cases refractory to standard chemotherapy treatment.

The possibility of bringing forward immunotherapy, with equal and positive results in more advanced cases of chemoresistance, where more chemotherapy regimens have been used, allows a treatment with a lower risk for immediate or late toxicity to be offered to these patients.^41^ This is potentially encouraging given successful reports of pregnancy subsequent to immunotherapy for GTN,^13^^,^^29^^,^^42^ making earlier initiation of this treatment feasible and potentially safe.

Patients with high-risk GTN with multidrug-resistant disease or relapse, especially those with exposure to etoposide above a cumulative dose of 2 g/m^2^ or those in whom treatment with chemotherapy containing multiple agents exceeds 6-months,^41^, ^42^, ^43^, ^44^ may especially benefit from pembrolizumab. The most established polychemotherapy regimens for the treatment of high-risk GTN contain etoposide (among which: EMA/CO, EP/EMA, TP/TE, escalated EP, high dose chemotherapy with Carbo-EC-T or ICE regimen – Supplemental Table 3).^1^^,^^2^^,^^4^ The possibility of sequencing the initiation of pembrolizumab after chemoresistance to one of these regimens is interesting, notably in cases of toxicity, without necessarily having to follow all these lines to use immunotherapy.^41^

The results of pembrolizumab for PSTT and ETT, equally good and comparable to choriocarcinomas, represent a new frontier for these tumors that are historically poorly chemo‐sensitive and which, in general, present greater lethality.^45^^,^^46^ It was especially interesting that the response to pembrolizumab extended beyond 4 years of disease which normally carries a dismal prognosis for cases of PSTT/ETT.^45^^,^^46^ In exceptional situations, where the standard treatment for these cases, hysterectomy, is not accepted by the patient due to her reproductive desire, the use of pembrolizumab as a fertility-sparing strategy has been successfully used in cases of PSTT (which potentially could also be applied to the ETT).^29^

The combination of immunotherapy with standard-of-care chemotherapy is already supported for a number of malignant diseases^47^ and appears to be safe and effective in GTN. Although anti-PD-1 therapy combined with chemotherapy in GTN has been reported to have shown improved antitumor effects and tolerable toxic effects, the immunotherapy used was camrelizumab (PD-1 inhibitor) combined with apatinib.^48^ Pembrolizumab associated with escalated EP, in an initial associated regimen, followed only by treatment with pembrolizumab facilitated the cure of a patient with choriocarcinoma with brain metastasis and chemoresistant to multiple lines of chemotherapy, with a disease-free survival of 14-months at the time of publication of the seminal case report of this regimen.^32^

This meta-analysis included studies from Europe, Asia, South America, and North America, indicating that this may allow the results to be globally generalizable. In addition, the involvement in this study of authors recognized as experts in GTN may enhance the conduct of the study and the interpretation of results. The main limitation of this paper is the rarity of GTN cases treated with immunotherapy, causing almost exclusively case reports to be included in this systematic review. However, there are already models and references for meta-analyses carried out exclusively with case reports, in the absence of RCTs or more robust studies, which is a common situation in rare diseases, which validates the present study in light of the available evidence in rare diseases.^49^^,^^50^ The authors should also emphasize the small number of cases in which there was no response to pembrolizumab, which may constitute a publication bias. This has a special impact on the meta-analysis of the evaluated subgroups, meaning that small samples in some subgroups may limit the power to detect significant differences. Furthermore, subgroup analyses were exploratory in nature and should be interpreted with caution. Future studies with larger samples and pre-specified subgroup analysis are needed to confirm these findings and further investigate possible predictors of treatment response. Another aspect that should be highlighted is the lack of some treatment details and the short follow-up time after remission that may have limited the diagnosis of GTN relapse, especially when evaluating the number of necessary consolidation cycles of pembrolizumab. Finally, it should be noted that the exclusion of articles written in a non-English language, as well as abstracts/presentations without full-text availability, is also a limitation of the study.

## Conclusions

Pembrolizumab seems to be an effective immunotherapy for the treatment of patients with high-risk GTN with chemoresistant or relapsed disease, including cases of PSTT and ETT. The good response to pembrolizumab was maintained in a wide range of patients, regardless of their age, origin of GTN, time to initiate immunotherapy, number of chemotherapy regimens used before immunotherapy, and whether or not it was associated with chemotherapy.

With its good clinical response, it is expected that pembrolizumab will be increasingly used in clinical practice in cases of high-risk GTN, PSTT, or ETT that do not respond to standard chemotherapy, as well as in relapsed diseases or in scenarios where, due to side effects or immediate or late toxicity to antineoplastics, a treatment with better tolerability is preferred. These results are promising and warrant further investigation in larger, prospective studies to confirm the efficacy of pembrolizumab in high-risk GTN.

## Authors’ contributions

MB, AB, MR, JMM, KE, NH and RB contributed to the conception of the study protocol and search strategy. MPR and AB designed the statistical analysis plan. The manuscript of the protocol was drafted by MB, AB, MR and JMM, and was critically revised by SYS, JRF, KE, NH and RB. AB, MR and JMM registered the protocol with the PROSPERO database. MB, AB, MR, JMM, KE, NH and RB analyzed the results and made the necessary clinical correlations. All authors wrote and approved the final version of the paper.

## Conflicts of interest

The authors declare that they have no known competing financial interests or personal relationships that could have appeared to influence the work reported in this paper.

## Appendix Supplementary materials

Image, application 1
