# Supplementary material for: Pembrolizumab in gestational trophoblastic neoplasia: Systematic review and meta-analysis with sub-group analysis of potential prognostic factors
Source: Clinics (Sao Paulo). 2025 Mar 2;80:100583. doi: 10.1016/j.clinsp.2025.100583 (PMC11923758; doi:10.1016/j.clinsp.2025.100583)
Supplement: Supplementary file 1 [file mmc1.docx]

**CLINICS-D-24-01382_Supplementary Material**

**Supplemental Figure 1** Quality assessment for case series and case reports using the Newcastle Ottawa scale.

**Supplemental Figure 2** Funnel plots and Egger’s test to visually assess publication bias.


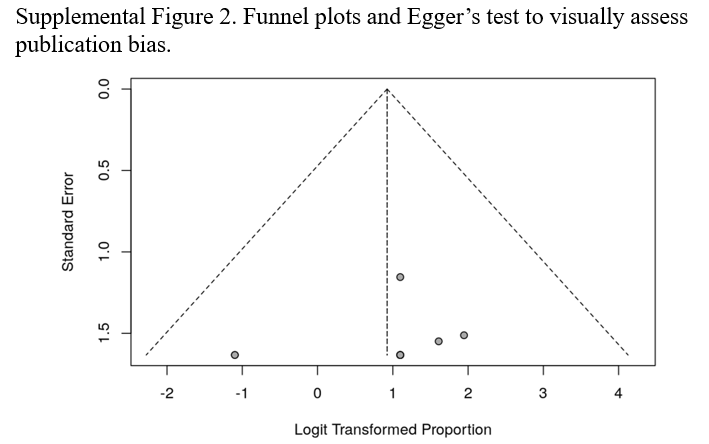


**Supplemental Table 1** Detailed searching strategy and results on immunotherapy for gestational trophoblastic neoplasia.

| **Database** | **Search Strategy** | **Number of papers outputted** |
| --- | --- | --- |
| PubMed | (("gestational trophoblastic Disease" [All Fields]) OR ("gestational trophoblastic neoplasia" [All Fields])) ("choriocarcinoma" [All Fields]) OR ("placental site trophoblastic tumor" [All Fields]) OR ("epithelioid trophoblastic tumor" [All Fields])) AND ("immunotherapy" [MeSH Terms]) AND (("remission" [All fields]) OR ("persistent" [All fields]) OR ("progression")) AND (("chemoresistant") OR ("refractory" [All fields]) OR ("resistant"[All fields]) OR ("non-respondent" [All fields]) AND ((“relapse” [All fields]) OR (“recurrence” [All fields])) | 164 |
| Scopus | (("gestational trophoblastic Disease" [All Fields]) OR ("gestational trophoblastic neoplasia" [All Fields])) ("choriocarcinoma" [All Fields]) OR ("placental site trophoblastic tumor" [All Fields]) OR ("epithelioid trophoblastic tumor" [All Fields])) AND ("immunotherapy" [MeSH Terms]) AND (("remission" [All fields]) OR ("persistent" [All fields]) OR ("progression")) AND (("chemoresistant") OR ("refractory" [All fields]) OR ("resistant"[All fields]) OR ("non-respondent" [All fields]) AND ((“relapse” [All fields]) OR (“recurrence” [All fields])) | 214 |
| Web of Science | (("gestational trophoblastic Disease" [All Fields]) OR ("gestational trophoblastic neoplasia" [All Fields])) ("choriocarcinoma" [All Fields]) OR ("placental site trophoblastic tumor" [All Fields]) OR ("epithelioid trophoblastic tumor" [All Fields])) AND ("immunotherapy" [MeSH Terms]) AND (("remission" [All fields]) OR ("persistent" [All fields]) OR ("progression")) AND (("chemoresistant") OR ("refractory" [All fields]) OR ("resistant"[All fields]) OR ("non-respondent" [All fields]) AND ((“relapse” [All fields]) OR (“recurrence” [All fields])) | 181 |
| Embase | (("gestational trophoblastic Disease" [All Fields]) OR ("gestational trophoblastic neoplasia" [All Fields])) ("choriocarcinoma" [All Fields]) OR ("placental site trophoblastic tumor" [All Fields]) OR ("epithelioid trophoblastic tumor" [All Fields])) AND ("immunotherapy" [MeSH Terms]) AND (("remission" [All fields]) OR ("persistent" [All fields]) OR ("progression")) AND (("chemoresistant") OR ("refractory" [All fields]) OR ("resistant"[All fields]) OR ("non-respondent" [All fields]) AND ((“relapse” [All fields]) OR (“recurrence” [All fields])) | 358 |

**Supplemental Table 2** Clinical and therapeutic overview of patients treated with pembrolizumab for gestational trophoblastic neoplasia.

| **Variables** | **Total (n = 21)** | **Remission (n = 18)** | **Non-remission (n = 3)** | **p-value** |
| --- | --- | --- | --- | --- |
| **Age** **(years)** |  |  |  |  |
| Median | 38 | 35.5 | 50 | 0.05^a^ |
| Quartiles (1^st^; 3^rd^) | 30; 46 | 28.8; 44.2 | 40; 51 |  |
| Range / SD | 23‒52 | 23‒49 | 38‒52 |  |
| **Histopathology** |  |  |  | 0.35^b^ |
| Choriocarcinoma | 9 (42.9%) | 7 (38.9%) | 2 (66.7%) |  |
| Mixed PSTT/ETT | 1 (4,8%) | 0 (0%) | 1 (33.3%) |  |
| PSTT | 4 (19%) | 4 (22.2%) | 0 (0%) |  |
| ETT | 4 (19%) | 4 (22.2%) | 0 (0%) |  |
| Invasive mole | 1 (4.8%) | 1 (5.6%) | 0 (0%) |  |
| Not available | 2 (9.5%) | 2 (11.1%) | 0 (0%) |  |
| **Origin of gestational trophoblastic neoplasia** |  |  |  | 0.50^b^ |
| After delivery | 6 (28.5%) | 6 (33.3%) | 0 (0%) |  |
| CHM | 3 (14.3%) | 3 (16,7%) | 0 (0%) |  |
| Abortion | 3 (14,3%) | 2 (11.1%) | 1 (33.3%) |  |
| Not available | 9 (42.9%) | 7 (38.9%) | 2 (66.7%) |  |
| **Time to innitiate immunotherapy (weeks)** |  |  |  |  |
| Median | 35 | 22 | 39 | 0.23^a^ |
| Quartiles (1^st^; 3^rd^) | 11.0; 73.0 | 9.0; 92.7 | 27.3; 166.5 |  |
| Range | 2‒204 | 2‒204 | 25‒192 |  |
| Not available | 1 | 1 | 0 |  |
| **hCG level pre immunotherapy** **(IU/L)** |  |  |  |  |
| Median | 118 | 38.1 | 2,468.0 | 0.07^a^ |
| Quartiles (1^st^;3^rd^) | 56; 2,291.5 | 1,474; 25,234 | 811.3; 40,411.3 |  |
| Range | 0‒48,000 | 0‒17,000 | 480‒48,000 |  |
| Not available | 2 | 2 | 0 |  |
| **Indication for immunotherapy** |  |  |  | 1.00^c^ |
| First line | 2 (9.52) | 2 (11.11%) | 0 |  |
| Resistance | 9 (42.86%) | 8 (44.44%) | 1 (33.33%) |  |
| Relapse | 10 (47.61%) | 8 (11.11%) | 2 (66.67%) |  |
| **Number of previous chemotherapy lines** |  |  |  |  |
| Median | 3 | 3 | 6 | 0.25^a^ |
| Quartiles (1^st^; 3^rd^) | 2.0; 4.0 | 1.9; 4.0 | 2.7; 6.0 |  |
| Range | 0‒6 | 0‒6 | 2‒6 |  |
| **Immunotherapy + Chemotherapy** |  |  |  |  |
| Yes | 2 (9.52) | 2 (100%) | 0 | 1.00^c^ |
| No | 19 (90.48%) | 16 (84.2%) | 3 (15.8%) |  |

^a^ Mann-Whitney *U* test.

^b^ Kruskal-Wallis test.

^c^ Chi-Square test: Immunotherapy + Chemotherapy.

**Supplemental Table 3** Polychemotherapy regimens for the treatment of gestational trophoblastic neoplasia containing etoposide.

| **Chemotherapy** | **Scheme** | | | |
| --- | --- | --- | --- | --- |
|  | **Day** | **Drug** | **Dose** | **Route** |
| EMA/CO | 1, 2 | Dactinomycin | 0.5 mg | IV Bolus |
|  | 1, 2 | Etoposide | 100 mg/m^2^ | IV |
|  | 1 | Methotrexate | 300 mg/m^2^ | IV |
|  | 2, 3 | Folinic Acid | 15 mg every 12 hours for 4 doses (to be started 24hrs after methotrexate) | PO |
|  | 8 | Vincristine | 0.8 mg/m^2^ (cap dose at 2 mg) | IV |
|  | 8 | Cyclophosphamide | 600 mg/m^2^ | IV |
| EP/EMA | 1 | Etoposide | 150 mg/m^2^ | IV |
|  | 8 | Etoposide | 100 mg/m^2^ | IV |
|  | 8 | Cisplatin | 75 mg/m^2^ | IV |
|  | 8 | Methotrexate | 300 mg/m^2^ | IV |
|  | 8 | Dactinomycin | 0.5 mg | IV Bolus |
|  | 9, 10 | Folinic Acid | 15 mg every 12 hours for 4 doses (to be started 24hrs after methotrexate) | PO |
| TP/TE | 1 | Paclitaxel | 135 mg/m^2^ | IV |
|  | 1 | Cisplatin | 60 mg/m^2^ | IV |
|  | 15 | Paclitaxel | 135 mg/m^2^ | IV |
|  | 15 | Etoposide | 150 mg/m^2^ | IV |
| Escalated EP | 1 | Etoposide | 250 mg/m^2^ | IV |
|  | 1 | Etoposide | 250 mg/m^2^ | IV |
|  | 1 | Cisplatin | 60 mg/m^2^ | IV |
| **High dose chemotherapy** |  | | | |
| Carbo-EC-T | -7 | Paclitaxel | 75 mg/m^2^ | IV |
|  | -7 | Etoposide | 450 mg/m^2^ | IV |
|  | -7 | Carboplatin | AUC 10 | IV |
|  | -5 | Paclitaxel | 75 mg/m^2^ | IV |
|  | -5 | Etoposide | 450 mg/m^2^ | IV |
|  | -5 | Carboplatin | AUC 10 | IV |
|  | -5 | Cyclophosphamide | 60 mg/kg | IV |
|  | -5 | Mesna | 120 mg/kg | IV |
|  | -3 | Paclitaxel | 75 mg/m^2^ | IV |
|  | -3 | Etoposide | 450 mg/m^2^ | IV |
|  | -3 | Carboplatin | AUC 10 | IV |
|  | -3 | Cyclophosphamide | 60 mg/kg | IV |
|  | -3 | Mesna | 120 mg/kg | IV |
|  | 0 | Reinfusion of peripheral bool stem cells | 2×10^6^ CD 34+ cells/kg | IV |
| ICE | 1‒3 | Etoposide | 100 mg/m^2^ | IV |
|  | 2 | Carboplatin | AUC 5 (cap dose at 800 mg) | IV |
|  | 2 | Ifosfamide | 5,000 mg/m^2^ | IV |
|  | 2 | Mesna | 5,000 mg/m^2^ | IV |
|  | 3 | Mesna | 2,000 mg/m^2^ at 2 and 6 hours post completion of ifosfamide | PO |
